# An Investigation of Fecal Volatile Organic Metabolites in Irritable Bowel Syndrome

**DOI:** 10.1371/journal.pone.0058204

**Published:** 2013-03-13

**Authors:** Iftikhar Ahmed, Rosemary Greenwood, Ben de Lacy Costello, Norman M. Ratcliffe, Chris S. Probert

**Affiliations:** 1 Department of Gastroenterology, University of Bristol/Bristol Royal Infirmary, Bristol, United Kingdom; 2 Department of Research and Development, Bristol Royal Infirmary, Bristol, United Kingdom; 3 Department of Applied Sciences, University of the West of England, Bristol, United Kingdom; 4 Department of Gastroenterology, Institute of Translational Medicine, University of Liverpool, Liverpool, United Kingdom; University of Chicago, United States of America

## Abstract

Diagnosing irritable bowel syndrome (IBS) can be a challenge; many clinicians resort to invasive investigations in order to rule out other diseases and reassure their patients. Volatile organic metabolites (VOMs) are emitted from feces; understanding changes in the patterns of these VOMs could aid our understanding of the etiology of the disease and the development of biomarkers, which can assist in the diagnosis of IBS. We report the first comprehensive study of the fecal VOMs patterns in patients with diarrhea-predominant IBS (IBS-D), active Crohn's disease (CD), ulcerative colitis (UC) and healthy controls. 30 patients with IBS-D, 62 with CD, 48 with UC and 109 healthy controls were studied. Diagnosis of IBS-D was made using the Manning criteria and all patients with CD and UC met endoscopic, histologic and/or radiologic criteria. Fecal VOMs were extracted by solid phase microextraction (SPME) and analyzed by gas chromatography-mass spectrometry (GC-MS). 240 VOMs were identified. Univariate analysis showed that esters of short chain fatty acids, cyclohexanecarboxylic acid and its ester derivatives were associated with IBS-D (p<0.05), while aldehydes were more abundant in IBD (p<0.05). A predictive model, developed by multivariate analysis, separated IBS-D from active CD, UC and healthy controls with a sensitivity of 94%, 96% and 90%; and a specificity of 82%, 80% and 80% respectively (p<0.05). The understanding of the derivation of these VOMs may cast light on the etiology of IBS-D and IBD. These data show that fecal VOMs analyses could contribute to the diagnosis of IBS-D, for which there is no laboratory test, as well as IBD.

## Introduction

IBS is a common functional gastrointestinal (GI) disorder, which accounts for up to 20% of gastroenterology referrals in the UK [1], [2] and approximately 4 million physician office visits in the USA annually [3]. The etiology of IBS is complex and poorly understood. It may be viewed as a multi-factorial disorder where dysregulation of the so-called brain–gut axis, alongside abnormal function in the enteric, autonomic and/or central nervous systems, causes symptoms [4]–[6]. However, recently the fecal microbiota of patients with IBS has been reported to differ from healthy controls [7].

IBS is a syndrome, characterized by gastrointestinal symptoms for which, as yet, there is no precise pathological explanation or biological markers. Often patients exhibit a predominant symptom of either diarrhoea or constipation in which case it is called diarrhoea-predominant (IBS-D) or constipation-predominant IBS (IBS-C); mixed IBS (IBS-M) is said to occur where there is a mixed pattern in which diarrhoea and constipation appear to alternate [8]. In current practice, most patients are diagnosed using the Manning [9] or the Rome criteria [10] in the absence of red flag symptoms (such as anemia, unintentional weight loss, rectal bleeding, a family history of bowel cancer) and exclusion of organic disease using endoscopy and radiological investigations [11].

In clinical practice it may be difficult to differentiate patients newly-presenting with either IBS-D or inflammatory bowel disease (IBD) [12]. Consequently, many physicians rely on invasive procedures in order to exclude IBD and other organic diseases. Furthermore, patients with IBS may harbor fears that their symptoms are due to serious pathology, especially when they experience severe pain. Such patients utilize significant healthcare resources by undergoing numerous investigations, frequent office visits and hospitalizations [13]. The use of invasive procedures exposes patients to their attendant risks and has a substantial economic impact. To give some indication of the scale of IBS, this condition costs the USA economy $10 billion per annum, in direct medical costs and a further $20 billion in indirect costs due to absenteeism and suboptimal productivity [14]; for Europe the cost is estimated to be €700 – €1600 per person per year [14], [15].

In order to distinguish between IBS and IBD, serological and fecal markers have been explored. C- reactive protein (CRP) and erythrocyte sedimentation rate (ESR) are popular tests, recommended by National Institute for Health and Clinical Excellence (NICE) UK guidelines, although they lack specificity. Fecal calprotectin and lactoferrin are more likely to reflect luminal pathology than serological markers and have been shown to help differentiate between IBD and IBS, when used in conjunction with symptoms based (Rome/Manning) criteria [16]–[19]. IBS is associated with normal investigations, but so is health. A technique that can support the diagnosis of IBS in a positive way may reassure patients and prevent numerous negative investigations.

Volatile organic metabolites (VOMs) are chemicals which may be emitted from the feces and contribute to its odor [20]–[22]. Fecal odor may change in the presence of GI disorders and understanding these changes may help in diagnosing various diseases. VOMs are generated by metabolism within the gut – partly by epithelium, partly by microbiota and partly from diet. Changes in the fecal VOMs may result from changes in the diet [23]; however, our group has reported that the majority of the fecal VOMs are shared by individuals and remain relatively constant in health with few changes due to day-to-day dietary habits [24]. Changes in VOMs may also result from pathology in the GI tract and/ or changes in the microbiota. Our group has also described the changes in fecal VOMs from patients with *Campylobacter*, *Clostridium difficile* and ulcerative colitis [24]; necrotizing enterocolitis [25] and cholera [26].

The hypothesis of the current study is that in IBS and IBD there might be specific changes in VOMs as a result of the changes in the fecal microbiota or changes in the intestinal epithelial chemistry. We analyzed the VOMs profile of headspace gases from feces of patients with IBS-D, Crohn's disease (CD), ulcerative colitis (UC) and healthy controls. We report, for the first time, that IBS-D can be differentiated from CD, UC and healthy controls on the basis of the fecal volatile organic metabolites.

## Methods

Four groups were studied; these were patients with IBS-D (n = 30), active CD (n = 62), active UC (n = 48), and healthy controls (n = 109). This study was designed to compare the fecal VOMs pattern of IBS-D with active IBD, as these are the main differential diagnosis of chronic diarrhoea in our clinical practice; therefore, cases with inactive CD and UC were not included. The median age was 42 yrs (19–78 yrs) with a male to female ratio of 1∶2. Diagnosis of IBS-D was made using the Manning criteria along with normal hematological investigations, negative celiac serology and gastrointestinal investigations, as judged appropriate by the clinician managing the patient. Manning criteria were developed in our unit over 30 years ago, unlike other criteria they are easily applied at initial consultation and do not require diary data. All patients with IBD had an endoscopic diagnosis with histological confirmation, except for patients with isolated small bowel CD where the site of disease was not accessible by endoscopy, in whom the diagnosis was made using radiology. Disease activity was established on the basis of clinical scoring criteria using Harvey Bradshaw Index (HBI) in the case of CD [27] and simple colitis clinical activity index (SCCAI) in the case of UC. A simple colitis clinical activity index (SCCAI) is a simple, easy and readily calculated index of disease activity using a small number of clinical criteria and does not depend on endoscopy assessment or laboratory indices. It scores from 0–15; a score of < 6 corresponds to mild disease, between 6–12 corresponds to moderate disease and score of >12 corresponds to severe disease. The SCCAI has been shown good correlation both with complex activity index for UC and Powel-Tuck score [28]. In this study we used SCCAI scoring for disease activity and our cut off value was 7 (mean = 11.4) for active UC; once again, these tools can be used at initial consultation without diary data. All patients with CD who had HBI scores of ≥ 4 (mean HBI score  = 10.7 in CD and mean SCCAI  =  11.0 in case of UC, Table 1) and those of UC with SCCAI scores of ≥7 along with raised inflammatory markers (mean CRP =  35.1 in CD and 30.8 in UC, Table 1) were classified as having active IBD. A record of medications was obtained for all participants. The demographic features and activity indices are summarized in Table 1.

**Table 1:** Demographics of the study participants.

	CD	UC	IBS	Healthy controls
Total No.	62	48	30	109
Sex	F = 32	F = 23	F = 23	F = 69
Age	18-80(Mean = 39)	18-77(Mean = 38)	19-65 (Mean = 24)	24-76 (Mean = 33)
Ethnic origin	Caucasian = 52	Caucasian = 39	Caucasian = 20	Caucasian = 99
	British Asian = 2	British Asian = 4	British Asian = 4	British Asian = 2
	Asian = 3	Asian = 1	Asian = 1	Asian = 4
	Others = 5	Others = 4	Others = 5	Others = 4
CRP (mg/dl)	Mean = 35.1(17-209)	Mean = 30.8(11-116)	NA	NA
Activity score	Mean = 10.7(4 -17)	Mean = 11.04(7-15)	NA	NA
Medications	Steroids 77%	Steroids 79%	Loperamide 33%	NA
	Azathioprine 45%	Azathioprine 38%	Mebevarin 60%	
	Methotrexate 10%	Methotrexate 0%	Buscopan 30%	
	5ASA 53%	5ASA 75%	Amitriptyline 20%	
	anti TNF 32%	anti TNF 8%	None 13%	

Ethical approval for the study was granted by the Wiltshire Research and Ethics Committee. Patients were recruited from outpatient clinics and the gastroenterology ward in the Bristol Royal Infirmary. Patient information sheets were provided to all study participants. Verbal consent was obtained after answering their queries and was documented in the clinical information sheets.

Local research and ethics committee approved the verbal consent process.

### Fecal samples

Fresh fecal samples were collected in 30 ml stool collection bottles (Nantong Shenhua Laboratorial Apparatus Co., Ltd. China) universally available and used for stool sample collection in NHS hospitals in the UK. From each sample, a 2 gm aliquot was placed into a 18 ml glass vial (Supelco, Sigma Aldridge, Poole, UK), sealed with a silicone/polytetraflouroethylene septum, within 6 hours of sample production and were frozen at −20°C until analyzed.

### GC-MS analysis

**Figure 1:**
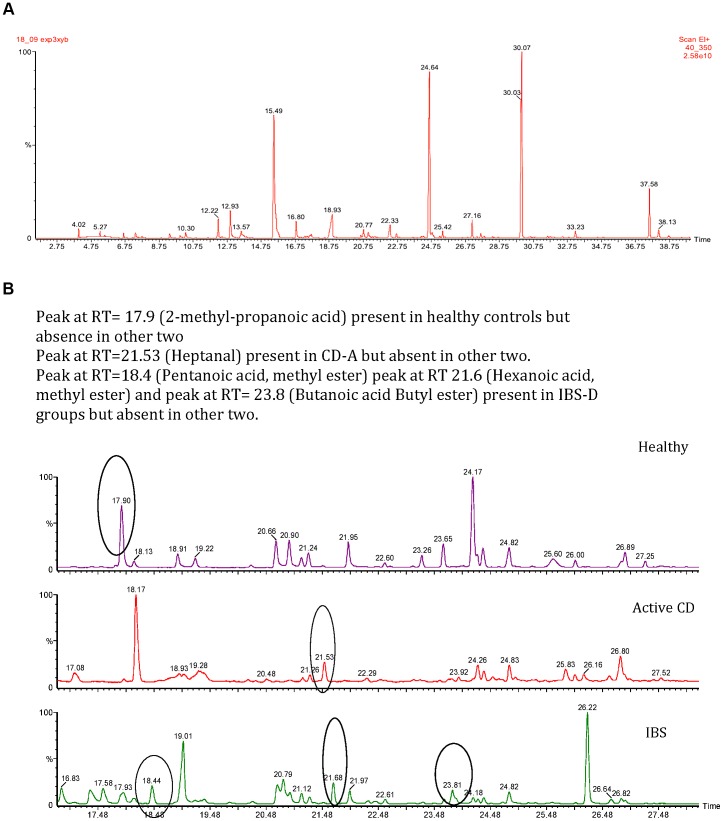
Figure 1A: A typical chromatogram from fecal headspace gas from a healthy volunteer. Figure 1B: A comparison of chromatograms from three different study groups (CD, IBS-D and healthy controls) showing absence and presence of VOMs peaks.

A detailed analytical method was developed to optimize the experimental conditions and was described previously [29]. We compared the efficiency of two types of solid phase microextraction (SPME) fibers polydimethylsiloxane/carboxane (PDMS/car) and divinylbenzene (DVB)] for their efficiency to extract a complex variety of VOMs from the feces; the PDMS/car fiber was found to be better in extracting a wide variety of VOMs from the feces and was used in this study. Before analysis, stored samples were placed with their lower third submerged in a water bath at 60°C for one hour. VOMs were then extracted using a preconditioned PDMS/car SPME fiber (Sigma Aldridge, Poole, UK) exposed to the headspace above the heated feces for a further 10 minutes. The fiber was then immediately transferred to the heated injection port (220°C) of a Clarus 500 (Perkin Elmer, Beaconsfield UK) GC-MS for thermal desorption. The GC-MS system was fitted with a 60 m×0.25 mm internal diameter SPB-1 column coated with a 1 µm film of stationary phase (Supelco, Poole, UK). The injector was equipped with a 1.5 mm quartz liner and operated in splitless mode. The oven temperature was held at 40°C for 2 minutes after the injection, and then heated up at 6°C/minute to 220°C, and held for 4 minutes giving a run time of 36 minutes. Pure helium (99.95%, BOC, Guilford, UK) was used as the carrier gas at a constant linear velocity of 35 cm/sec. A typical chromatogram with the peaks representing individual compounds is shown in Figure 1A.

The MS was operated in electron ionization mode scanning a mass range 17–350 with the filament emission current set at 200 mA. The ionization waveform was set ‘on’. The ion trap was operated at a target value of 50, a trap offset of 10 V and at a sampling rate of 2 scans/sec. The multiplier was set at 3.9×10^5^. The ion trap manifold temperature was set at 180°C and the transfer line was 220°C. Ethanol standards (50 ppm, BOC, Guilford, UK) were used to ensure the SPME fiber efficiency daily [29].

Each chromatogram generated was analysed for identification of compounds. The chromatograms were integrated and a search criterion for peak identification was set at peak area of 1,000,000. Fecal VOMs were identified by comparing the fragment pattern with those in the National Institute of Standard and Technology 2008 (NIST) library with a set match criteria of more than 90% followed by manual visual inspection using retention time matching of selected standards (Fisher Scientific, Loughborough, UK; Acros Organics, London, UK; Sigma-Aldrich, UK) where needed. There were small numbers of peaks for which the NIST library search did not find a correct match. This was either because the compound was not in the NIST library or may be because two or more different compounds co-eluted making correct assignation very challenging. Such unidentified peaks (approximately 30 in total in all experiments) were named as unknown compounds as per their retention time (for example “unknown compound RT-30.8” for peak appeared at 30.8 minutes) and these unknown compounds were also included in the analysis as unknown compounds. All chromatograms were re-inspected for the presence of sub-threshold peaks and compounds were recorded where a match was available after background subtraction.

### Statistical methods

Data analysis was carried out using SPSS (Statistical Package for Social Sciences, version 16). The characteristics of the study subjects are shown in the Table 1.

240 VOMs were identified in our study subjects and each compound was assigned a value of 1 or 0 based on its presence or absence. Using binary data (i.e. presence or absence of compounds), univariate analysis was performed to identify key VOMs at a significant level (p<0.05), which might contribute to the model to discriminate between the groups. A multivariate discriminant function analysis was performed using these key volatiles in a forward stepwise entry manner to develop a discriminatory model to discern differences between the groups. The results obtained by this analysis were then cross validated using *leave–one–out* cross validation and multi-step split sample approach. Cross-validation is a statistical way of assessing how the results of a statistical analysis will generalize to an independent data set and how accurately a prediction model will perform in practice [30]. In *leave-one-out* cross-validation, the predictive model was redeveloped using all but one case from the data set, that one case being omitted temporarily. The group membership of the omitted case was then predicted using the model and the accuracy of the prediction was recorded. This process was repeated for each case in the data one by one. For multi-step split sample cross-validation the data was divided using an 80∶20 split, the first set (80% of the data called the training data set) was used to redevelop the model, the accuracy of which was then tested on the remaining 20% of the data (validation data set). This process was repeated 10 times and each time a different set of training data and validation data was selected randomly to assess the stability of the predictive model. Split sample cross-validation is a method for estimating generalization error based on re-sampling and the purpose of this split sample validation in multivariate analysis is to reduce the possibility of over-fitting and of non-reproducible results.

## Results

A total of 240 VOMs were identified in these experiments. The mean number of VOMs per CD case was 74 (range = 34–125), per UC case was 70 (range = 25–101), per IBS case was 88 (range = 42–113) and per healthy control was 90 (range = 50–128). An average of 2 VOMs per case were found to be unique to that case and were not detected in any other case. These VOMs appeared to be person-specific and were excluded from the analysis. Univariate analysis showed no significant difference in the fecal VOMs due to sex and ages of the groups.

### Analysis of IBS-D, CD and UC

Univariate analysis identified 44 key fecal VOMs which were significant (p<0.05) in separating IBS-D from CD and UC. 35 VOMs were significantly more abundant in IBS-D group, 6 VOMs in the CD groups and three in UC group (Table 2). Esters of short chain fatty acids, cyclohexanecarboxylic acid and its derivatives were significantly associated with IBS-D groups, aldehydes and ketones were associated with CD groups while the three VOMs more abundant in UC were (1-propanol, 2-methyl), (undecane) and (methoxy-phenyl-oxime). These key VOMs were used to develop a discriminatory model using multivariate discriminant function analysis in a forward step-wise manner as stated above. A comparison of significant compounds in chromatograms from patients with IBS, active IBD and healthy controls is shown in Figure 1B.

**Table 2:** Statistically significant VOMs in three groups (p<0.05).

VOMs abundant in IBS-D	VOMs abundant in CD	VOMs abundant in UC
Pentanoic acid	Heptanal	1-Propanol, 2-methyl-
Butanoic acid, methyl ester	Propanal	Undecane
Pentanoic acid, methyl ester	Pentanal	Methoxy-phenyl-oxime
Butanoic acid, butyl ester	2-Heptanone, 6-methyl-	
Butanoic acid, 3-methyl-, propyl ester	S-Methyl 3-methylbutanethioate	
Hexanoic acid, methyl ester	2-Piperidinone	
Acetic acid, butyl ester		
Propanoic acid, butyl ester		
Butanoic acid, 2-methylpropyl ester		
Cyclohexanecarboxylic acid, ethyl ester		
Butanoic acid, 3-methyl-methyl ester		
Cyclohexanecarboxylic acid, methyl ester		
Acetic acid, pentyl ester		
Butanoic acid, 2-methyl-, propyl ester		
Butanoic acid, 3-methyl-, butyl ester		
Propanoic acid, 2-methyl-, methyl ester		
Propanoic acid, 2-methylpropyl ester		
Cyclohexanecarboxylic acid, propyl ester		
Pentanoic acid, butyl ester		
Cyclohexanecarboxylic acid, butyl ester		
Benzoic acid, 2-hydroxy-, methyl ester		
Butanoic acid, 2-methylbutyl ester		
Cyclohexanecarboxylic acid		
1-methyl-2-(1-methylethyl)-benzene		
1,4-Cyclohexadiene, 1-methyl-4-(1-methylethyl)-		
5-methyl-2-(1-methylethyl)-cyclohexanol		
Copaene		
Pentanoic acid, 4-methyl-		
compound-95 (RT-30.8)		
á-Pinene		
Phenol, 4-methyl-		
1-Butanol, 3-methyl-, propanoate		
2-Butanol, (ñ)-		
Methyl alcohol		
á-Phellandrene		
Ethylbenzene		

### IBS-D vs. CD

The discriminatory model was able to correctly identify 100% of CD cases and 80% of IBS-D cases, which on *leave-one-out* cross-validation reduced to 97% and 80% (p = 0.001) respectively showing the stability of the model (Table 3).

**Table 3:** Classification results of IBS, active IBD, active CD, active UC and healthy controls analyses.

Analysis	Groups	Number	Correctly identified	Cross validated	p value
IBS vs. active IBD	IBS	30	80%	70%	0.002
	Active IBD	110	96%	95%	
IBS vs. CD	IBS	30	80%	80%	0.001
	Active CD	62	100%	97%	
IBS vs. UC	IBS	30	87%	83%	0.001
	Active UC	48	94%	92%	
IBS vs. Healthy controls	IBS	30	70%	68%	<0.05
	Healthy controls	109	95%	94%	

**Figure 2:**
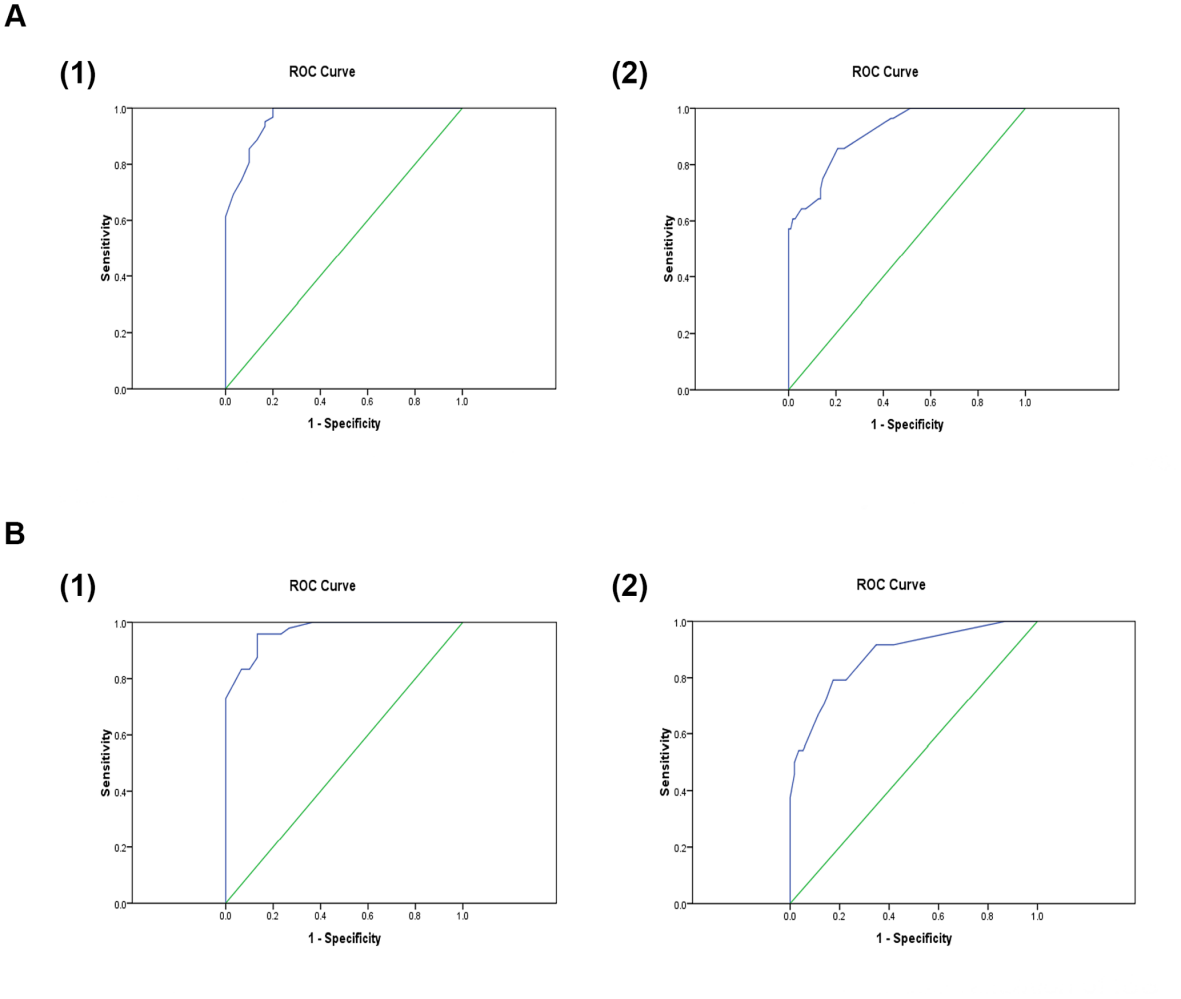
Figure 2A1: Statistical analysis of IBS vs. CD. AUC = 0.97. 2: Cross-validation of IBS vs. CD analysis. AUC = 0.93. Figure 2B1: Statistical analysis of IBS vs. UC. AUC = 0.96. Figure 2B2: Cross-validation of IBS vs. UC analysis. AUC = 0.88.

The receiver operator characteristic (ROC) curve was created for this analysis and observed area under the curve (AUC) was 0.97 showing a diagnostic sensitivity of the model of 94% and specificity of 82% in separating IBS-D from CD. This model was further validated using split sample cross-validation and repeated 10 times. The average AUC of these 10 cross validation analyses was 0.93 showing the diagnostic sensitivity of the model to be 90% if the specificity is set at 80% (Figure 2A1, 2A2).

### IBS-D vs. UC

The discriminatory model was able to correctly identify 87% of IBS-D cases from UC and 94% of the UC cases correctly, which on *leave-one-out* cross-validation reduced to 83% and 92% respectively (p = 0.001) showing the stability of the model (Table 3).

The AUC for the ROC curve was 0.96 showing a diagnostic sensitivity of 96% and specificity of 80%. The AUC on split sample cross-validations analyses reduced to 0.88. This model has diagnostic sensitivity of 90% if the specificity is set at 80% (Figure 2B1, 2B2).

### Analysis of IBS-D vs. active IBD

In this analysis, CD and UC cases were grouped together as active IBD and compared with IBS-D. 60 VOMs were identified by univariate analysis; 50 were found to have a positive association with IBS-D (p<0.05) and the other 10 VOMs were positively associated with active IBD (Tables 4 and 5). VOMs belonging to the aldehydes class (Table 5) were more commonly identified in the active IBD group (p<0.05).

**Table 4:** VOMs positively associated with IBS compared with active IBD.

Compounds	IBS (%)	Active IBD (%)	p value
1-Butanoic acid, methyl ester	90	71	0.02
Methyl alcohol	90	67	0.009
Propanoic acid, methyl ester	87	66	0.002
Pentanoic acid, methyl ester	77	51	0.009
Caryophyllene	70	50	0.04
1-Methyl-2-(1-methylethyl)-benzene	70	34	0.001
Butanoic acid, propyl ester	70	40	0.003
Butanoic acid, butyl ester	67	40	0.008
Hexanoic acid, methyl ester	63	17	0.000
1-Methyl-4-(1-methylethyl)-1,4-cyclohexadiene	60	29	0.002
Copaene	53	30	0.017
Acetic acid, butyl ester	53	24	0.002
Butanoic acid, 3-methyl-, butyl ester	53	18	0.00
Butanoic acid, 2-methyl-, propyl ester	50	23	0.004
á-Phellandrene	50	22	0.003
Propanoic acid, butyl ester	50	20	0.002
Cyclohexanecarboxylic acid, ethyl ester	50	16	0.00
Bicyclo [3.1.1] 6,6-dimethyl-2-methylene-heptane	47	28	0.046
1-Methyl-4-1-methylethylidene-cyclohexene	47	19	0.003
Cyclohexanecarboxylic acid, methyl ester	47	13	0.000
á-Pinene	47	17	0.001
Butanoic acid, 3-methyl-, propyl ester	43	22	0.019
Unknown compound RT-8.2	43	15	0.002
Cyclohexanecarboxylic acid, propyl ester	43	14	0.001
Acetic acid, pentyl ester	43	10	0.000
Pentanoic acid, butyl ester	43	10	0.000
2-Hexanone	40	22	0.04
Butanoic acid, 2-methylpropyl ester	40	14	0.003
Cyclohexanecarboxylic acid	37	17	0.02
Propanoic acid, l, 3-methyl-1-butyl ester	37	13	0.004
6-Methyl- 5-hepten-2-one	33	16	0.04
Butanoic acid, 2-methyl-, methyl ester	33	14	0.02
Propanoic acid, 2-methyl-, methyl ester	33	14	0.016
2-Butanol, (ñ)-	33	9	0.04
Propanoic acid, 2-methylpropyl ester	30	13	0.028
1,6-Octadien-3-ol, 3,7-dimethyl-2-aminobenzoate	30	12	0.02
5-Methyl-2-(1-methylethyl)-cyclohexanol	27	9	0.017
á-Myrcene	27	6	0.004
Cyclohexanecarboxylic acid, butyl ester	27	5	0.002
1-Methyl-4-(1-methylethyl)-1,3-cyclohexadiene	27	5	0.002
Benzoic acid, 2-hydroxy-, methyl ester	23	6	0.012
Heptanoic acid, methyl ester	20	7	0.05
Butanoic acid, 3-methyl-, methyl ester	20	7	0.05
Butanoic acid, 2-methylbutyl ester	20	6	0.034
5-Methyl-2-(1-methylethyl)-cyclohexanone	20	6	0.034
Butanoic acid, 1-methylethyl ester	20	4	0.007
Unknown compound RT-12.9	20	4	0.013
Pentanoic acid, 2-methylpropyl ester	20	4	0.013
Disulfide, methyl 2-propenyl	17	4	0.037
Propanoic acid, hexyl ester	13	3	0.038

**Table 5:** Compounds positively associated with active IBD compared with IBS.

Compounds	Active IBD (%)	IBS (%)	p value
2-Methylpropanal	84	67	0.04
Undecane	77	57	0.024
Heptanal	75	47	0.003
3-Methylbutanal	70	50	0.035
Isopropyl alcohol	47	20	0.005
2-Methyl,1-propanol	44	23	0.027
Cyclohexene	22	4	0.000
Methoxy-phenyl-oxime	20	3	0.02
Butanoic acid, 3-methyl-S-methyl ester	19	3	0.025
Butanoic acid, 2-methyl-, ethyl ester	18	3	0.032

**Figure 3:**
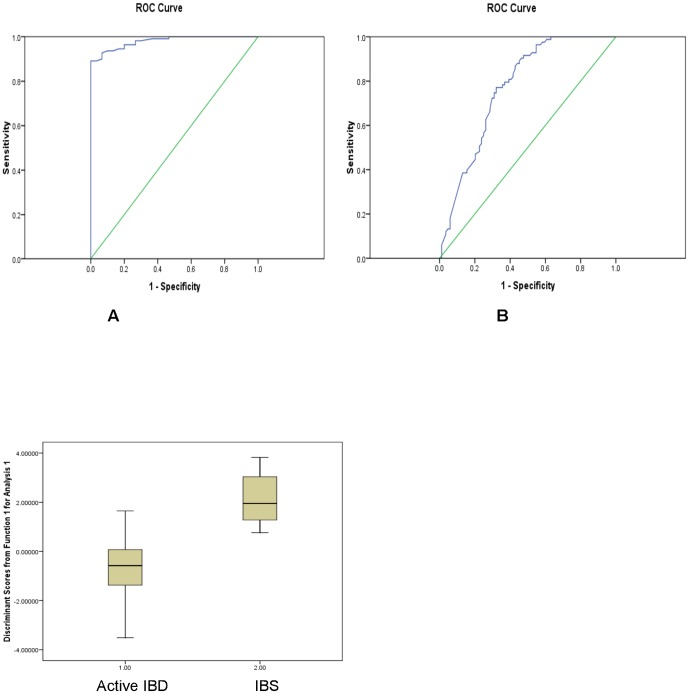
Box and Whisker plot of discrimination of IBS from active IBD.

The discriminatory model, which was developed by multivariate analysis using key VOMs (Table 6), identified 80% of IBS-D cases and 96% of active IBD cases correctly, which fell to 70% and 95% respectively on *leave-one-out* cross-validation (p =  0.002). The ROC curve for this analysis showed that the sensitivity of the model was 96% and a specificity of 80%, AUC value of 0.98 (Table 3, Figure 3A).

**Table 6:** Discriminatory model of IBS vs. active IBD based on presence and absence of fecal volatiles in the two groups.

Steps	Compounds	Statistics	Df1	Df2	Sig.
1	Hexanoic acid, methyl ester	.819	1	1	.000
2	1-Methyl-2-(1-methylethyl)-benzene	.732	2	1	.000
3	á-Myrcene	.687	3	1	.000
4	Heptanal	.642	4	1	.000
5	Unknown compound RT-8.2 min	.607	5	1	.000
6	Methyl alcohol	.572	6	1	.000
7	Butanoic acid, 3-methyl-S-methyl ester	.548	7	1	.000
8	2-Hexanone	.526	8	1	.000
9	Propanoic acid, 3-methyl-1-butyl ester	.495	9	1	.000
10	5-Methyl-2-(1-methylethyl)-cyclohexanol	.477	10	1	.000
11	Undecane	.458	11	1	.000

On further revalidation using multi-steps split sample approach using a 80∶20 split and 10 times repetitions, the average value for AUC for the ten cross-validation analyses was 0.76 (Figure 3B). This showed that the model was stable in separating the IBS-D from the active IBD group with a sensitivity of 80% and specificity of 62%.

### Statistical analysis of IBS-D vs. healthy controls

In this analysis, the fecal VOMs profiles of IBS-D patients (n = 30) were compared with healthy controls (n = 109). Using binary data (presence or absence), univariate analysis identified 49 VOMs at a significant level (p<0.05). There were 28 VOMs which were significantly (p<0.05) more abundant in IBS-D and of these, 22 VOMs belong to the ester class showing its strong association with IBS-D (Table 7). However, no distinctive pattern of VOMs was found to be positively associated with healthy controls; ketones, aldehydes and organic acids were each found to have a weak association with this group (Table 8). The discriminatory model (Table 9) developed by multivariate analysis correctly identified 95% of healthy controls and 70% of IBS cases respectively which reduced to 94% and 68% respectively on *leave-one-out* cross-validation (p = <0.05) (Table 3).

**Table 7:** VOMs positively associated with IBS compared with healthy controls.

Compounds	IBS (%)	Controls (%)	p value
Butanoic acid, ethyl ester	90	72	0.034
Propanoic acid, methyl ester	87	70	0.047
1-Methyl-2-(1-methylethyl)-benzene	70	39	0.003
Butanoic acid, butyl ester	67	44	0.048
Butanoic acid, propyl ester	70	32	0.001
Hexanoic acid, methyl ester	63	36	0.007
Propanoic acid, propyl ester	50	28	0.011
Acetic acid, butyl ester	53	24	0.006
Butanoic acid, 3-methyl-, butyl ester	53	11	0.000
Propanoic acid, butyl ester	50	24	0.015
Cyclohexanecarboxylic acid, ethyl ester	50	21	0.006
Butanoic acid, 2-methyl-, propyl ester	50	9	0.00
Ethanoic acid, ethyl ester	43	27	0.042
Pentanoic acid, 4-methyl	40	22	0.042
Acetic acid, pentyl ester	43	21	0.033
Pentanoic acid, butyl ester	43	19	0.02
Butanoic acid, 3-methyl-, propyl ester	43	17	0.005
Cyclohexanecarboxylic acid, propyl ester	43	15	0.004
6-Methyl-5-hepten-2-one	33	13	0.029
Propanoic acid, 3-methy1-butyl ester	37	8	0.001
Ethanoic acid, 3-methyl-1-butyl ester	27	7	0.004
Cyclohexanecarboxylic acid, butyl ester	27	5	0.004
Benzoic acid, 2-hydroxy-, methyl ester	23	5	0.013
Pentanoic acid, 4-methyl-, pentyl ester	20	5	0.013
Butanoic acid, 3-methyl-, methyl ester	20	4	0.007
Thiopivalic acid	17	5	0.038
5-Methyl-2-(1-methylethyl)-cyclohexanone	20	5	0.038
4-Methyl-1-Indole	17	3	0.12

**Table 8:** VOMs positively associated with healthy controls compared with IBS.

Compounds	IBS (%)	Controls (%)	p value
2-Heptanone	83	97	0.012
2-Methylpropanal	67	88	0.008
3-Methylbutanoic acid	67	84	0.032
Undecane	57	79	0.015
3-Methylbutanal	50	75	0.003
2-Methylpropanoic acid	40	69	0.004
2-Methyl-1-propanol	23	43	0.037
1R-à-Pinene	10	27	0.042
2-Pentylfuran	7	30	0.011
Methoxy-phenyl-oxime	3	27	0.000
2-Methylfuran	7	23	0.034

**Table 9:** Discriminatory model for the differentiation of IBS from healthy controls.

Steps	Compounds	Statistics	Df1	Df2	Sig.
1	Butanoic acid, 3-methyl-, butyl ester	.836	1	1	.000
2	Methoxy-phenyl-oxime	.768	2	1	.000
3	Butanoic acid, 3-methyl-, methyl ester	.711	3	1	.000
4	Cyclohexanecarboxylic acid, butyl ester	.661	4	1	.000
5	Undecane	.621	5	1	.000
6	2-Pentyl-furan	.582	6	1	.000
7	1R-à-Pinene	.542	7	1	.000
8	5-methyl-2-(1-methylethyl)-cyclohexanone	.515	8	1	.000
9	2-Methylpropanoic acid	.498	9	1	.000

**Figure 4:**
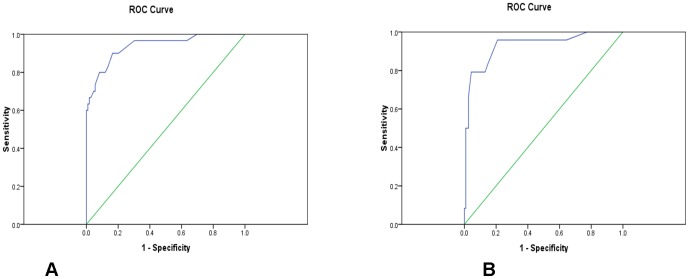
Figure 4A: ROC curve for IBS vs. controls. Figure 4B: ROC curve for cross-validation.

The ROC curve was created for this analysis (Figure 4A) and an observed AUC was 0.94, showing that the model could differentiate IBS-D from healthy controls with a sensitivity of 90% and a specificity of 80%. On further re-validation using the multi-steps split sample approach the AUC value for the ROC of these ten cross-validation analyses was 0.92 (Figure 4B), showing a diagnostic sensitivity of 82% and a specificity of 78%.

## Discussion

Our study has, for the first time, reported the analysis of volatile organic metabolites in headspace gases emitted from the feces of patients with IBS-D in comparison with those of active IBD and healthy individuals. Clear differences in the VOMs patterns were found that could distinguish between IBS and the other three groups.

Our study found that esters of short chain fatty acids, cyclohexanecarboxylic acid and its derivatives were present in increased abundance in the feces of IBS-D patients compared with active IBD, while fecal aldehydes, such as heptanal and propanal, were more abundant in active CD (p<0.05). The most commonly observed esters were the methyl esters of propanoic acid, butanoic acid, pentanoic acid and hexanoic acid.

Kajander *et al* [31] investigated the mucosal metabolic profile of IBS in comparison with healthy controls using gas chromatography-time of flight-mass spectrometer; they observed a 14-fold increase in the abundance of the cyclic ester 2(3H)-furanone in mucosal biopsies of the left colon of IBS patients along with an increase in several other lipids species. Interestingly, some organic acids (dodecanoic, azelaic and adipic acid) were found to be slightly reduced in abundance while decanoic acid was slightly increased in IBS patients compared to healthy controls, the author did not specify whether their study group consisted of patients with IBS-D or whether patients with constipation predominant IBS or mixed-IBS were included.

Studies have also reported increased level of organic acids in correlation with symptoms in IBS. For example, the study by Tana *et al* [32] has demonstrated a significantly higher level of organic acid such as acetic acid and propionic acid in the feces of patient with IBS in comparison with healthy controls using high performance liquid chromatography (HPLC). Their study group consisted of both IBS-D and mixed IBS; and increased levels of organic acids in IBS patients were found to be positively associated with severity of the symptoms. Importantly, neither of these manuscripts reported the IBD patients.

Many studies have suggested that organic acid and esters metabolites in the feces are correlated with the changes in the gut microbiota and their interaction with the dietary substrates. For example study by Tana et al [32] observed the altered profile of intestinal microbiota, especially *Lactobacilli* and *Veillonella*; and unbalanced fecal organic acid levels in IBS. *Lactobacilli* produce lactic acid and acetic acid from glucose and fructose by a fermentation process [33], while *Veillonella* transform lactic acid into acetic acid and propionic acid [34]. The data showing altered composition of gut microbiota in IBS is mounting and studies are needed to explore the relationship of this altered microbiota with fecal VOMs in correlation with symptoms of IBS.

Aldehydes are the product of lipid peroxidation and their concentration is increased in the breath and blood of patients in various inflammatory conditions. Lipid peroxidation is a degenerative process affecting the polyunsaturated fatty acids in the cell membrane that leads to the formation of lipid hydroperoxides. These hydroperoxides further decompose to generate a wide variety of carbonyl containing metabolites that are excreted in various bodily fluids including breath and the feces depending upon the site of inflammation. Our observation of higher abundance of aldehydes in the IBD group compared with the IBS-D suggests these VOMs are inflammation-related and not a result of disturbed microbiota or a change of pH as a result of diarrhea.

Secondly, our results also showed differences in the fecal volatile pattern of IBS in comparison with healthy individuals. In addition to higher abundance of esters in the IBS group, some organic acids were found in higher abundance while others were less frequently observed (Table 6 & 7). This observation is consistent with the study by Kajander *et al* [31].

Diet has been considered an important causative factor for various symptoms of IBS by affecting gut transit time, interaction with gut microbiota and fecal gas production [35], [36]. Studies have shown the effect of various type of diet (such as fiber-rich or fiber-free diet, low or high fermentable fiber diet) on gut transit time and colonic physiology [37], [38] and these changes in the colonic physiology can in turn affect the fecal VOMs. It is noteworthy that in most of these studies, dietary modifications were observed by the participants for a prolonged period (>1 week) in order to produce any measurable effects on colonic physiology. Recently, studies have shown that the foods high in FODMAP (Fermentable oligo, di, monosaccharides and polyoles) are responsible for triggering the symptoms of IBS and dietary intervention with foods low in FODMAP are shown to reduce the symptoms of IBS in particular abdominal pain and flatulence. Diets low in FODMAP are also shown to reduce the colonic gas production both in IBS and healthy controls [39]. [40]. Patients with IBS and those with IBD may change their dietary pattern frequently in response to their disease type in order to help their symptoms, and often do not remember what they ate in the past few days hence the lack of detailed dietary data in our study groups. It is therefore difficult to comment if any of the changes observed in the fecal VOMs of our study groups could be assigned to changing dietary pattern. This could be considered a weak point of the current study. In order to answer this important question, detailed dietary information on day-to-day basis and its correlation with the fecal VOMs would be of significant value.

Studies have suggested that breath hydrogen and methane can be used to assess GI conditions, such as small intestinal bacterial overgrowth (SIBO), IBS and IBD. Methane excretion was found to be associated with alterations in intestinal motility, higher methane excretion favoring constipation [41]–[43]. Our technique did not detect methane. However, long chain alkanes such as pentane, hexane, octane and decane were identified from the fecal samples but none of these appeared to be discriminatory between the groups. Short chain hydrocarbons (excluding methane) have been reported in breath as a result of inflammatory conditions generally and significant elevation of pentane levels have been reported in patients with inflammatory bowel diseases when compared to healthy controls [44].

More recently fecal biomarkers such as fecal calprotectin and lactoferrin have shown promising results in differentiating IBD from IBS, monitoring disease activity and predicting the relapse in IBD [45], [46]. Disappointingly, these are non-specific markers of bowel inflammation as their levels are also abnormal in NSAID-induced bowel inflammation, celiac sprue, colonic polyps, diverticulitis and colorectal cancer [47], [48]. Moreover, the predictive value of fecal calprotectin for clinical relapse was poor in case of CD compared to UC [49], [50]. There was no difference in the value of fecal calprotectin in IBS patients compared to healthy controls which shows that these markers can only be used to differentiate organic disease from functional bowel disorders such as IBS but cannot diagnose IBS from healthy controls [51]. On the other hand, our studies demonstrated that fecal VOMs could separate IBS from healthy individual with a sensitivity of 90% and a specificity of 80%. However, a comparison of these currently available fecal biomarkers with fecal volatile metabolite would be of great clinical interest and warrant further studies as these fecal biomarkers in conjunction with each other may provide more detailed solution of IBS-IBD differentiation challenge than in isolation.

Differentiation of IBS from inactive IBD is, perhaps, of less clinical concern than IBS differentiation from active IBD. Neither CRP nor fecal markers are shown to be of any value in discriminating IBS from inactive IBD. Our data in the differentiation of IBS from inactive IBD, as expected, did not show impressive results (data not shown). These results imply that the change in the fecal VOMs, in particular the aldehyde pattern, was due to inflammation; and in the absence of such inflammation (in case of inactive IBD), the aldehyde markers are reduced or disappear. The aim of the current study was to compare the changes in the fecal VOMs pattern of IBS with active IBD. Data comparing CD and UC with due consideration to disease distribution and severity are beyond the scope of this benchmark paper.

Medications are considered to have an effect on the composition of fecal VOMs which may either be due to the direct effects of medications on the fecal microbiota and altered absorption of various nutrients or due to excretion of its metabolites in the fecal stream [52]. Patients in this study were maintained on different classes of medications for the treatment of IBD, which were recorded and correlated with the changes in the volatile profiling. The majority of patients with active disease were on IV or oral steroids, 5ASA medications or immunosuppressive drugs. A small number of participants with CD were also on biological agents. Similarly in the IBS category, the majority of patients were either on mebeverine or loperamide (Table 1). Our study did not detect any salicylic acid or any active or inactive metabolites of medications in the feces of study participants; however, it remains difficult to decide if medications have any impact on fecal VOMs profiles. To understand the influence of these medications on fecal VOMs, controlled trials in the future would be of great clinical value.

In short, with the improvements in sample preparation techniques and availability of modern technique such as GS-MS, the field of volatile organic metabolomes is evolving rapidly. It is already considered a sensitive analytical tool for investigating the health-disease continuum. Various small studies now have reported the analysis of volatile pattern of breath, urine and blood in the diagnosis and monitoring of various inflammatory and malignant conditions with encouraging results [53]–[56]. The characteristic patterns of VOMs in feces have also been reported for diverse causes of diarrhea [24]. Any method, however, has its limitations. Large numbers of fecal metabolites are included in the studies and caution is necessary in the interpretation of results with specific attention being required to consider the possibility of contamination of the results by the environment [57]. The desired discovery of a single distinctive biomarker might not be possible, but systematic changes in the abundance of specific groups of molecules (such as certain esters, organic acids and aldehydes in the current study) indicate a biologically relevant volatile metabolite type. A drawback of fecal VOMs analysis is that a large proportion of spectral peaks are still unknown, and consequently more effort has to be invested in the compilation of standardized metabolite libraries. Considering the current study setting, a further weakness is the small number of subjects. Nevertheless, it is encouraging to see that IBS-D patients were differentiated from active IBD and healthy controls with high sensitivity, and that the developed models were stable under cross-validation.

Moreover, it would have been interesting to investigate whether differences between IBS and IBD could also be observed in metabolic profiles from other non-invasive tissues, such as blood and urine as these are more easily obtained in clinical settings.

To summarize, the study of fecal VOMs analysis may provide the basis for the development of a simple, fast and convenient method of diagnosing various gastrointestinal disorders. Such techniques may be useful as a non-invasive diagnostic tool which can be performed repeatedly. Distinct changes were observed in the fecal VOMs pattern in IBS, IBD and healthy controls and further studies are warranted to explore in depth the relationship of these fecal VOMs with diet and gut microbiota. This may help in understanding the etiology of various GI conditions and the diagnostic value of these biomarkers in the clinical field. In the future, it is likely that the advent of more sensitive analytical techniques may lead to the development of a simple sensor technique, which can be specifically designed to identify the set of these clinically distinctive VOMs, and may provide a much-needed reliable, real-time and point-of-care diagnosis and monitoring of various gastrointestinal diseases.
